# Effects of Hydroxypropyl-Beta-Cyclodextrin on Cultured Brain Endothelial Cells

**DOI:** 10.3390/molecules27227738

**Published:** 2022-11-10

**Authors:** Szilvia Veszelka, Mária Mészáros, Gergő Porkoláb, Ágnes Rusznyák, Katalin Szászné Réti-Nagy, Mária A. Deli, Miklós Vecsernyés, Ildikó Bácskay, Judit Váradi, Ferenc Fenyvesi

**Affiliations:** 1 Institute of Biophysics, Biological Research Centre, Eötvös Loránd Research Network, Temesvári krt. 62, H-6726 Szeged, Hungary; 2 Doctoral School of Biology, University of Szeged, Dugonics tér 13, H-6720 Szeged, Hungary; 3 Department of Pharmaceutical Technology, Faculty of Pharmacy, University of Debrecen, Nagyerdei St. 98, H-4032 Debrecen, Hungary; 4 Doctoral School of Pharmaceutical Sciences, University of Debrecen, H-4032 Debrecen, Hungary; 5 Institute of Healthcare Industry, University of Debrecen, Nagyerdei St. 98, H-4032 Debrecen, Hungary

**Keywords:** blood–brain barrier, 2-hydroxypropyl-beta-cyclodextrin, endocytosis, Niemann–Pick disease, type C

## Abstract

The application of 2-hydroxypropyl-beta-cyclodextrin (HPBCD) in the treatment of the rare cholesterol and lipid storage disorder Niemann–Pick disease type C opened new perspectives in the development of an efficient therapy. Even if the systemic administration of HPBCD was found to be effective, its low permeability across the blood–brain barrier (BBB) limited the positive neurological effects. Nevertheless, the cellular interactions of HPBCD with brain capillary endothelial cells have not been investigated in detail. In this study, the cytotoxicity, permeability, and cellular internalization of HPBCD on primary rat and immortalized human (hCMEC/D3) brain capillary endothelial cells were investigated. HPBCD shows no cytotoxicity on endothelial cells up to 100 µM, measured by impedance kinetics. Using a fluorescent derivative of HPBCD (FITC-HPBCD) the permeability measurements reveal that on an in vitro triple co-culture BBB model, FITC-HPBCD has low permeability, 0.50 × 10^−6^ cm/s, while on hCMEC/D3 cell layers, the permeability is higher, 1.86 × 10^−5^ cm/s. FITC-HPBCD enters brain capillary endothelial cells, is detected in cytoplasmic vesicles and rarely localized in lysosomes. The cellular internalization of HPBCD at the BBB can help to develop new strategies for improved HPBCD effects after systemic administration.

## 1. Introduction

Niemann–Pick disease, type C (NPC) is a fatal, lysosomal cholesterol and lipid storage disorder, with neurological symptoms and visceral involvement [1]. The intracellular transport of cholesterol is inhibited due to the mutation of the *NPC1* or *NPC2* genes, causing the accumulation of unesterified cholesterol in lysosomes and late endosomes. Significant alterations of glycosphingolipids were also observed in the brain. One of the most promising treatments of NPC is using cyclodextrins [2,3]. The systemic administration of 2-hydroxypropyl-beta-cyclodextrin (HPBCD) reduced the cholesterol and ganglioside accumulation in neurons and significantly increased the lifespan of both Npc1^−/−^ and Npc2^−/−^ mice [4]. The positive neurological effects of subcutaneous or intraperitoneal administration of HPBCD were unexpected, as HPBCD was considered to have low permeability through the blood–brain barrier (BBB). The major limiting factor of the effectiveness of HPBCD in the central nervous system (CNS) after systemic administration is the low BBB permeation [5]. Indeed, the systemic administration of HPBCD was changed to intrathecal administration and proved to be more effective. The result of a non-randomized, open-label, phase 1–2 trial showed that lumbar intrathecal HPBCD slowed disease progression with an acceptable safety profile [6]. However, systemic administration is more convenient to the patients than intrathecal administration. Nanoparticles containing beta-cyclodextrin (BCD) can be a promising way to improve the bioavailability of CD in the central nervous system of mice [7]. CD-based macromolecular systems [8,9], or improving the complexation capacity by polymerization [10], can enhance the efficacy of intravenously administered HPBCD, but the exact mechanism of the transport of HPBCD through the BBB is still undiscovered. Recent studies in mice show that [^14^C]-HPBCD has an important interaction with BBB; significant [^14^C]-HPBCD binding to the brain vasculature without crossing of the blood–brain barrier was revealed [11]. In another study, the4 majority of the [^14^C]-HPBCD loosely adhered to the luminal surface of brain endothelial cells without evidence of sequestration by capillaries. Only a small amount crossed the BBB by a non-saturable mechanism consistent with transcellular diffusion [12]. This phenomenon has not been investigated at cellular level yet, even though the endocytosis of cyclodextrins was revealed earlier [13,14]. On the other hand, the intracellular fate of HPBCD is another question. A recent study reported that significant amount of rhodamine-labeled HPBCD and random methyl-β-cyclodextrin (RAMEB) was detected in lysosomes after internalization in Caco-2 cells [15]. Other results show that HPBCD and (2-Hydroxypropyl)-γ-cyclodextrin (HPGCD) mobilize cholesterol from late endosomes/lysosomes [16], especially via the lysosome-associated membrane protein 1 (LAMP-1) [17]. Furthermore, HPGCD improved the autophagic functions of NPC1 patient-derived fibroblasts, resulting in the improvement in cellular homeostasis [18]. In the treatment of the NPC mouse model, equal efficacy, but significantly reduced toxicity, was found in case of HPGCD compared with HPBCD [19]. According to another study, the presence of suitable extracellular acceptors is required for the cyclodextrin-dependent cholesterol exchange from the plasma membrane in U2OS cells [20]. It can be hypothesized that the endocytosis of HPBCD can take place in the endothelial cells of BBB, therefore, the aim of this study was to examine the permeability and internalization of HPBCD on primary rat brain endothelial cells and compare it to immortalized human hCMEC/D3 cells. For this, the fluorescently labelled HPBCD derivative (FITC-HPBCD) was used on the in vitro BBB models.

## 2. Results

### 2.1. Effect of HPBCD on Viability of Brain Endothelial Cells

**Figure 1:**
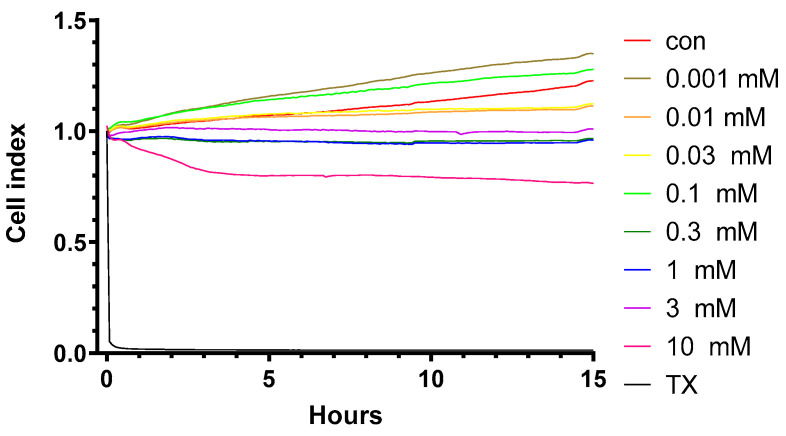
Kinetics of the effect of HPBCD on the viability of primary rat brain endothelial cells monitored by impedance measurement.

**Figure 2:**
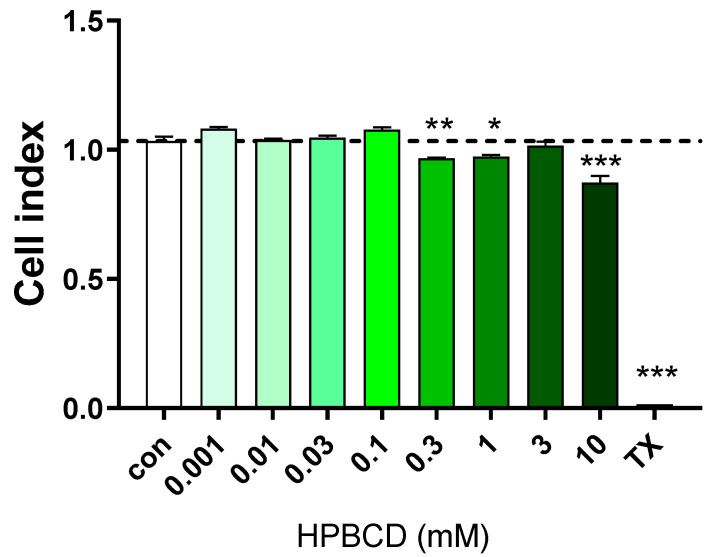
Effect of 2 h treatment with HPBCD on the viability of primary rat brain endothelial cells. Values presented are means ± SEM. Statistical analysis: one-way analysis of variance followed by Dunnett’s post-test. * *p* < 0.05, ** *p* < 0.01, *** *p* < 0.001 compared to control group, n = 6.

HPBCD does not change the impedance of primary rat brain endothelial cell monolayers in the range of 0.001–0.1 mM concentrations, but above 0.3 mM (300 µM) concentration, a decrease in the cell index values indicates cellular toxicity (Figure 1 and Figure 2). Triton X-100 detergent (10 mg/mL) was used as a reference compound, inducing cell toxicity [21,22].

**Figure 3:**
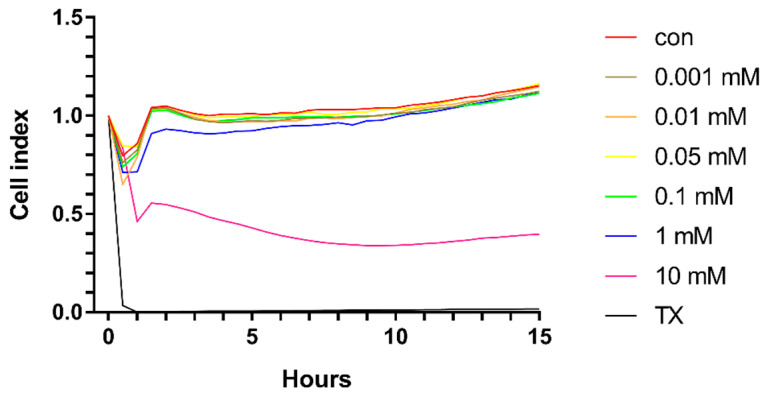
Kinetics of the effect of HPBCD on the viability of hCMEC/D3 cells monitored by impedance measurement.

**Figure 4:**
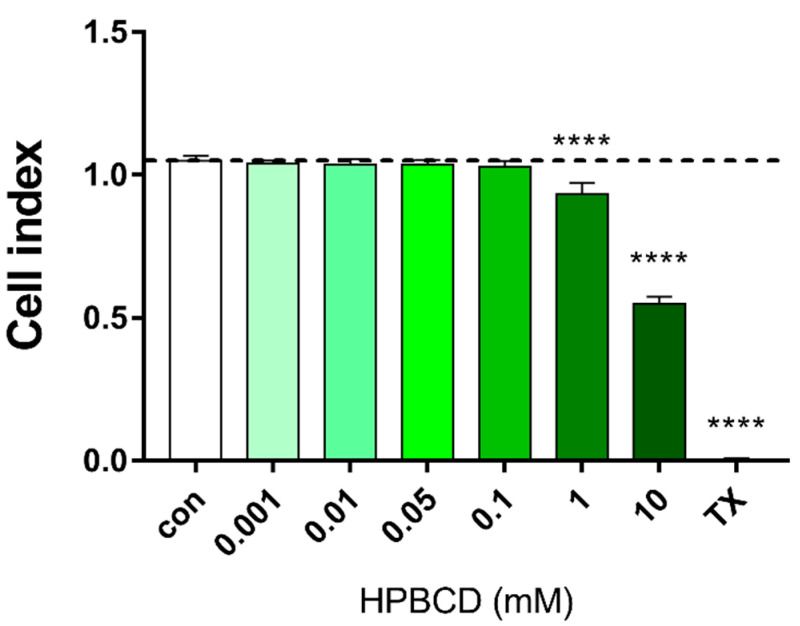
Effect of 2 h treatment with HPBCD on the viability of hCMEC/D3 cells. Values presented are means ± SEM. Statistical analysis: one-way analysis of variance followed by Dunnett’s post-test. **** *p* < 0.0001 compared to control group, n = 4 (except in case of 0.05 and 0.01 groups, where n = 3).

Similarly, in the primary rat brain endothelial cells, HPBCD does not decrease the cell index of hCMEC/D3 monolayers in the range of 0.001–0.1 mM (Figure 3 and Figure 4). Based on the previous study of Fenyvesi et al. [13], the 50 µM concentration of HPBCD was selected to test in BBB permeability studies, which can be considered as a safe concentration based on the toxicity results after 2 h of incubation (Figure 2 and Figure 4).

### 2.2. Uptake of FITC-HPBCD in Brain Endothelial Cells

**Figure 5:**
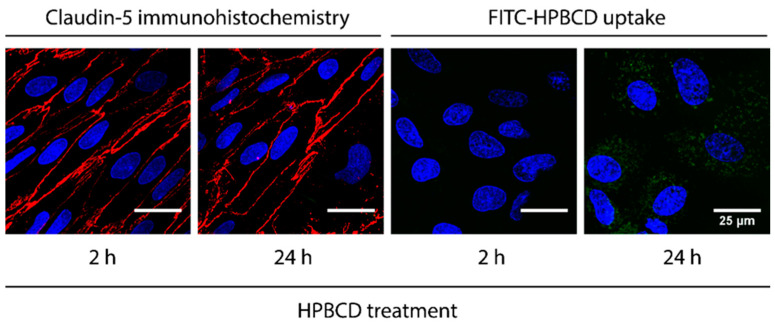
Visualization of the claudin-5 immunostaining (red) on fixed brain endothelial cells after HPBCD treatment (50 µM, 2 and 24 h) and the uptake of HPBCD (green) in living brain endothelial cells. Blue: cell nuclei. Bar: 25 µm.

The uptake of 50 µM FITC-HPBCD after 2 h treatment was visualized in primary rat brain endothelial cells by confocal microscopy (Figure 5). The green fluorescent signal is stronger after 24 h treatment monitored in living cells. Claudin-5 staining is concentrated to the cell border at the interendothelial junctions of fixed brain endothelial cells after 2 h treatment. The 24 h treatment with FITC-HPBCD results in the loss of continuous staining pattern in the claudin-5 staining (Figure 5).

**Figure 6:**
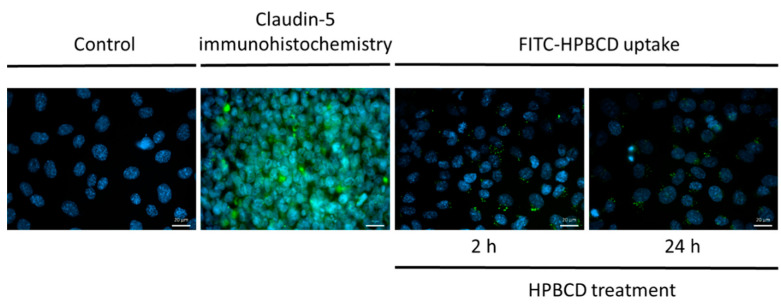
Fluorescence microscopic images of FITC-HPBCD-treated and untreated hCMEC/D3 cells. (Green: claudin-5 immunostaining on immunohistochemistry image and FITC-HPBCD on FITC-HPBCD uptake image; blue: cell nuclei) Bar: 20 µm.

The cellular internalization of FITC-HPBCD on hCMEC/D3 cells was visualized by fluorescence microscopy (Figure 6). Cyclodextrin-loaded vesicles are detected in the cytoplasm after 2 h and 24 h treatments. Claudin-5 immunostaining of hCMEC/D3 monolayers shows only intracellular presence of the tight-junction protein, therefore, the effect of HPBCD on claudin-5 intercellular connections at the cell borders could not be investigated (Figure 6).

### 2.3. Permeability of FITC-HPBCD across the Culture Models of the BBB

**Figure 7:**
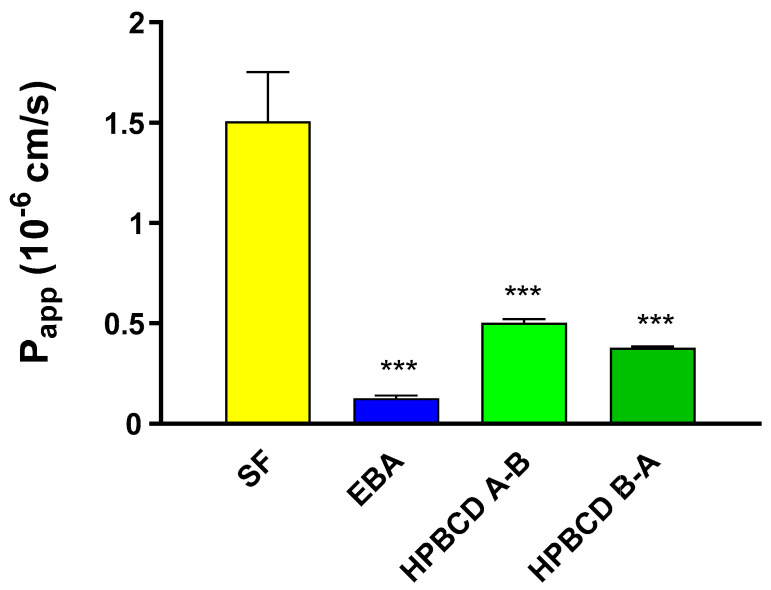
Permeability of FITC-HPBCD (50 µM) and marker molecules (SF, EBA) across the primary-cell-based BBB model. Values presented are means ± SEM. Statistical analysis: one-way analysis of variance followed by Dunnett’s post-test. *** *p* < 0.001, compared to SF treated group, n = 4. (P_app_-apparent permeability coefficient).

The barrier property of the BBB co-culture model using primary rat brain endothelial cells was sufficiently tight for the permeability assay based on the P_app_ values for the marker molecules (SF: 1.5 × 10^−6^ cm/s; EBA: 0.12 × 10^−6^ cm/s, Figure 7) in accordance with our previous studies [23,24]. The permeability coefficients of FITC-HPBCD in the A–B direction are 0.50 × 10^−6^ and in the B–A direction are 0.37 × 10^−6^ cm/s (Figure 7). These values indicate a low penetration of FITC-HPBCD across the rat BBB model in both directions, between the two permeability markers.

**Figure 8:**
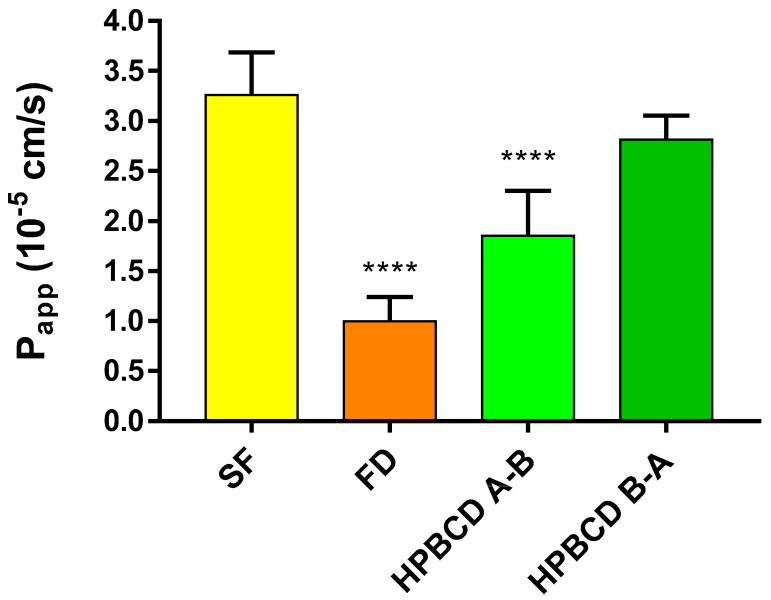
Permeability of FITC-HPBCD (50 µM) and marker molecules (SF, FD) on hCMEC/D3 cells. Values presented are means ± SEM. Statistical analysis: one-way analysis of variance followed by Tukey’s multiple comparisons post-test. **** *p* < 0.0001, compared to SF treated group, n for SF (A-–B) = 8; FD (A–B) = 12; F-HPBCD (A–B) = 8; F-HPBCD (B–A) = 4. (P_app_—apparent permeability coefficient).

In the case of the model using hCMEC/D3 cells, different permeability values are recorded compared to the primary rat brain endothelial cells. This model is more permeable to each of the molecules, however there are differences among the permeability values according to the molecular weights. The small molecule SF has the highest permeability value of 3.26 × 10^−5^ cm/s, while FD shows the lowest value, 1.0 × 10^−5^ cm/s. The apparent permeability of FITC-HPBCD is 1.86 × 10^−5^ cm/s and 2.82 × 10^−5^ cm/s in the A–B and B–A directions, respectively (Figure 8.).

### 2.4. Uptake of FITC-HPBCD by hCMEC/D3 Cells

**Figure 9:**
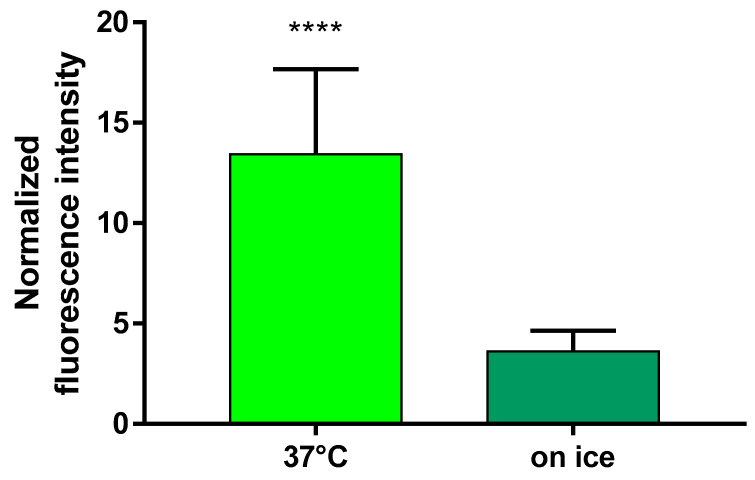
Uptake of FITC-HPBCD by hCMEC/D3 cells. Fluorescence intensity values of the samples are normalized to the value of the untreated control and presented as means ± SEM. Statistical analysis: unpaired t test with Welch’s correction (two-tailed *p* value). **** *p* < 0.0001, n = 12 for ‘37 °C’ and n = 13 for ‘on ice’.

To further analyze the cellular uptake of FITC-HPBCD, flow cytometric analysis was performed on hCMEC/D3 cells at 37 °C and keeping the cells on ice. hCMEC/D3 cells take up significantly higher amount of FITC-HPBCD at 37 °C than upon cooling, indicating an active cellular uptake process of the cyclodextrin (Figure 9).

### 2.5. Investigation of the Lysosomes

#### 2.5.1. Fluorescence Microscopy

**Figure 10:**
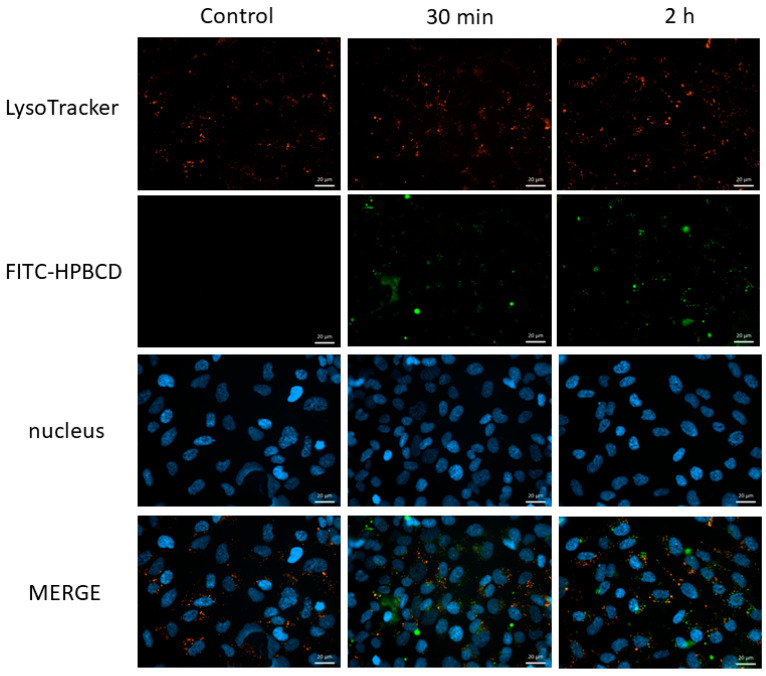
Fluorescence microscopic images of FITC-HPBCD containing intracellular vesicles and lysosomes in hCMEC/D3 cells after 30 min and 2 h of incubation. Blue: cell nuclei, green: FITC-HPBCD, red: lysosomes stained with Lysotracker, yellow pixels: colocalization of FITC-HPBCD and lysosomes. Bar: 20 µm.

The intracellular localization of FITC-HPBCD after internalization into hCMEC/D3 cells was investigated by simultaneous fluorescence staining of lysosomes, in order to reveal the intracellular fate of cyclodextrins. Figure 10 shows that FITC-HPBCD can be detected in intracellular vesicles (green) after 30 min or 2 h of incubation, but only a smaller fraction of vesicles colocalize (yellow) with lysosomes (red) even after 2 h of incubation. 

#### 2.5.2. Flow Cytometry

**Figure 11:**
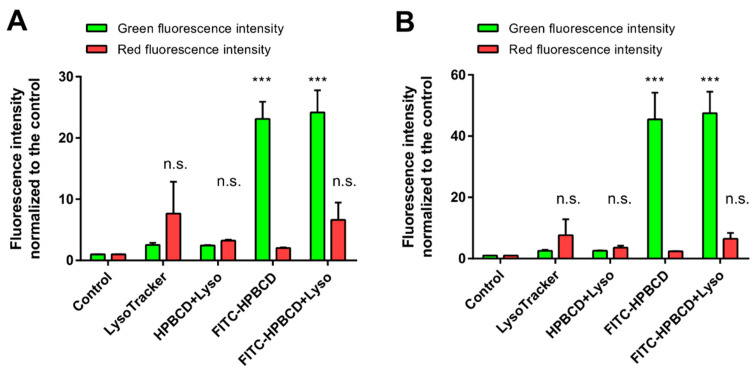
Results of flow cytometry analysis of lysosomal fluorescence of hCMEC/D3 cells after 30 min (**A**) or 2 h (**B**) of HPBCD and FITC-HPBCD incubation at 50 µM concentration. Red lysosomal fluorescence does not increase significantly after HPBCD or FITC-HPBCD treatments compared to untreated cells (n.s; *p* > 0.05 for both molecules). FITC-HPBCD accumulation significantly increases green cellular fluorescence compared to control cells (*** *p* < 0.001). Red fluorescence: LysoTracker, green fluorescence: FITC-HPBCD. Values are presented as means ± S.D. n = 4.

The formation of lysosomes in hCMEC/D3 cells was further investigated by flow cytometry after 30 min or 2 h of incubation with cyclodextrins (Figure 11). In this experiment, both FITC-HPBCD and unlabeled HPBCD were applied to have proper controls for the fluorescence measurements. The red fluorescence of lysosomes does not increase significantly after 30 min or 2 h of HPBCD or FITC-HPBCD incubation (*p* > 0.05 for both molecules), compared only to the LysoTracker-stained control. The green cellular fluorescence of FITC-HPBCD is approximately twice as high after 2 h of incubation than after 30 min, indicating the continuous cellular uptake of cyclodextrins. The comparison of the red fluorescence intensities of LysoTracker at the different time points reveals that there is no significant difference between the 30 min and 2 h values of lysosomal fluorescence (*p* > 0.05 for both HPBCD and FITC-HPBCD). The longer incubation of cells with HPBCD or FITC-HPBCD does not increase the number or the activity of lysosomes.

## 3. Discussion

Enormous effort was made to develop efficient drugs of NPC in recent years. Until now, the number of available treatments was limited, and the attention was mainly focused on HPBCD. Earlier, HPBCD was used as a pharmaceutical excipient to improve drug solubility and bioavailability, but the effectiveness of this cyclodextrin in NPC shed light on new strategies in the treatment of cholesterol storage disorders and neurological diseases related to cholesterol [25]. Presumably, the effectiveness of HPBCD in the treatment of NPC is based on the complexation of cholesterol and immobilization from intracellular pools. However, depending on the site of administration, HPBCD must at least overcome the barrier of the cell membrane in order to reach the sequestered cholesterol in late endosomes/lysosomes. After intravenous administration, HPBCD must also overcome the BBB to develop its positive neurological effects. There are different theories to explain the positive effect of HPBCD on BBB [4], but the interaction of this cyclodextrin with the brain capillary endothelial cells regarding its cellular internalization has not been examined yet. At first, the cytotoxicity of HPBCD was tested on primary rat brain endothelial cells and human hCMEC/D3 cells, and it was found that up to 100 µM HPBCD has no cytotoxicity. This was in accordance with earlier findings, where HPBCD was non-toxic on immortalized murine microvascular endothelial cells of the blood–brain barrier [26]. To study the permeability of HPBCD, the fluorescently labeled derivative (FITC-HPBCD) was used on the in vitro triple co-culture blood–brain barrier model, and also the hCMEC/D3 model. According to the results, FITC-HPBCD has low permeability on the rat BBB model, which agrees with the in vivo findings [11,12] and the results obtained with the in vitro BBB model with HPBCD [27]. Nevertheless, hCMEC/D3 monolayers show much less TEER and higher apparent permeability of HPBCD, even if the different permeabilities of marker molecules correspond to their molecular weight and show a functional barrier. The weak barrier property is in agreement with the low expression of claudin-5 tight-junction protein on hCMEC/D3 cells, while in primary rat brain endothelial cells, claudin-5 is well visible on the cell borders [28]. The permeability of fluorescein measured on hCMEC/D3 monolayers in an earlier study of Veszelka et al. is comparable to the presented results, showing the weak barrier properties of this cell line [28]. In this study, the cellular internalization of FITC-HPBCD was also investigated in both cell lines. According to the results, after 24 h incubation, a significant amount of FITC-HPBCD could be detected in the intracellular vesicles of primary rat brain endothelial cells, while in hCMEC/D3 cells, the green vesicles could be detected in endosomes after 30 min and the process could be inhibited by cooling. This is in accordance with previous findings, where Caco-2 cells internalize the fluorescence derivatives of HPBCD and RAMEB within 30 min of incubation [13,15,29], and in human umbilical vein endothelial cells (HUVECs), FITC-HPBCD could be detected too [30]. Interestingly, the cellular fluorescence measured by flow cytometer is different in the above-mentioned cell lines, indicating the different ability of cell types to internalize cyclodextrins. The possible explanation of the intracellular localization of FITC-HPBCD in the brain endothelial cells is that FITC-HPBCD enters the cells by endocytosis. This mechanism can also explain the unusual in vivo behavior of HPBCD at the BBB, by binding to the brain vasculature [11] or initiating the transcellular diffusion through endothelial cells [12]. The intracellular fate of cyclodextrins in brain-derived endothelial cells has not been examined yet. For the further investigations of lysosomes, only hCMEC/D3 cells were used, because of the limited availability of primary rat brain endothelial cells, while, on the other hand, this cell type shows the rapid internalization of FITC-HPBCD. In this study, the localization of FITC-HPBCD in late endosomes/lysosomes was investigated and it was found that only some lysosomes contained FITC-HPBCD on the fluorescence images of hCMEC/D3 cell layers. There is also a great difference compared to Caco-2 cells, where a significant amount of FITC-HPBCD localizes in lysosomes after internalization [15]. The internalization of cyclodextrins does not increase the fluorescence of lysosomes, indicating that probably neither the number of lysosomes or the lysosomal activity are increased by internalized HPBCD in the brain endothelial cells. In NPC1 mutant cells, HPBCD-treatment significantly increases the expression of lysosome-associated membrane protein 1 [17], indicating that the regulation of fine molecular mechanisms of cholesterol transport can be influenced and the mechanism of action of HPBCD is complex. On the other hand, the pathophysiology of NPC is also complex, and requires new combinative therapies with HPBCD. An alternative combination of VEGF overexpression in the brain and HPBCD systemic administration shows a synergistic effect and improvement in the pathophysiology of NPC [31].

In conclusion, the reported mechanism in this study can open new routes for drug development. Even if HPBCD cannot permeate the BBB in large amounts, it may enter the endothelial cells and start the cholesterol mobilization at the BBB. Modified HPBCD derivatives may have more efficient cellular internalization and better permeability. By applying more effective HPBCD derivatives, the intravenous doses could also be decreased in the treatment of neurological diseases [25]. On the other hand, further investigations are needed to reveal the exact internalization mechanism and its role in cholesterol mobilization at the BBB.

## 4. Materials and Methods

### 4.1. Reagents

All reagents were purchased from Merck (Budapest, Hungary), unless otherwise indicated. HPBCD and 6-deoxy-6-[(5/6)-fluoresceinylthioureido]-(2-hydroxypropyl)-β-cyclodextrin (FITC-HPBCD) were the products of Cyclolab Ltd. (Budapest, Hungary). FITC-HPBCD product number: CY-F-2005.1, average degree of substitution (DS) determined by NMR was DS = 0.7 for FITC and DS = 4.1 for hydroxypropyl group. FITC-HPBCD was purified by extensive dialization to remove impurities. Free dye and 6-deoxy-6-monoamino-HPBCD contents were tested by capillary electrophoresis (CE) and were below limit of detection. Stock solution of the cyclodextrin was prepared in sterile distilled water, from which treatment solutions were made in culture medium.

### 4.2. Cell Cultures

Isolation of rat brain endothelial cells, glia, and pericytes, and the construction of the in vitro BBB model, was established according to the method described in our previous studies [23,24]. After isolation, cells were cultured on dishes (Corning, Costar, New York, NY, USA) coated with 100 μg/mL collagen type IV and 100 μg/mL fibronectin in sterile distilled water. Endothelial cells were cultured in DMEM/HAM’s F-12 (Gibco, Life Technologies, Carlsbad, CA, USA), 15% plasma-derived bovine serum (PDS, First Link, Wolverhampton, UK), 100 μg/mL heparin, medium supplement with 5 μg/mL insulin, 5 μg/mL transferrin, 5 ng/mL sodium selenite (ITS, Pan-Biotech Gmbh, Germany), 1 ng/mL basic fibroblast growth factor (bFGF, Roche, Basel, Switzerland), and 50 μg/mL gentamycin. During the first three days, the culture medium of endothelial cells contained 3 μg/mL puromycin to eliminate P-glycoprotein negative, contaminating cell types [32]. For the triple co-culture BBB model, in addition to brain endothelial cells, primary rat brain pericytes and glial cell cultures were performed. Pericytes were isolated using the same method as brain endothelial cells, except that pericytes were cultured in uncoated dishes in DMEM/HAM’s F-12 supplemented with 10% fetal bovine serum (FBS, Pan-Biotech GmbH, Germany) and did not receive puromycin. Primary glial cells were prepared from one-day-old Wistar rats, as previously described [23], and passaged to 12 well plates (Corning, Costar, New York, NY, USA) coated with collagen type IV (100 μg/mL in sterile distilled water). Rat glial cells were cultured in DMEM/HAM’s F-12 supplemented with 10% FBS for two weeks before using them for the triple-culture BBB model.

The human immortalized hCMEC/D3 cells (Merck KGaA, Darmstadt, Germany, Cat. # SCC066) were cultured in EndoGRO-MV Complete Culture Media (supplemented with the components of the kit and fibroblast growth factor 2 (FGF-2) at 1 ng/mL final concentration). Cell culture flasks and the membranes of culture inserts were coated with 1:20 diluted collagen type I in phosphate-buffered saline (PBS).

### 4.3. Measurement of Cellular Toxicity

Kinetics of the reaction of rat brain endothelial cells to the HPBCD treatment were monitored by impedance measurement at 10 kHz (xCELLigence RTCA-SP instrument; Agilent, Santa Clara, CA, USA). Impedance measurement is a label-free, real time, non-invasive method, and correlates linearly with adherence, growth, number, and viability of cells [22]. For background measurements, 50 µL cell culture medium was added to the wells, then cells were seeded at a density of 5 × 10^3^ cells/well to rat tail collagen-coated 96-well plates with integrated gold electrodes (E-plate 96, ACEA Biosciences, San Diego, CA, USA). Cells were cultured for 4 days and monitored every 5 min until the end of experiments. At the beginning of plateau phase of growth, cells were treated with HPBCD (0.001–10 mM) for 15 h. Cell index was defined as R_n_-R_b_ at each time point of measurement, where R_n_ is the cell–electrode impedance of the well when it contains cells, and R_b_ is the background impedance of the well with the medium alone.

To determine the cytotoxicity of different concentrations of HPBCD on hCMEC/D3 cells an RTCA-DP Instrument (XCelligence system, ACEA Biosciences Inc., San Diego, CA, USA) was used. In this experiment, 1 × 10^4^ hCMEC/D3 cells/well were seeded on E-plates. After 3 days of incubation and reaching of plateau phase of growth, cells were treated with HPBCD solutions in different concentrations. The control group received culture medium. For both cell types, Triton-X100 was used as a reference agent to induce cell death. Cells were incubated with the solutions for 72 h and the cell index was measured every hour. Values were expressed as normalized cell index calculated by the software of the instrument.

### 4.4. Establishment of the In Vitro Triple Co-Culture Blood–Brain Barrier Model

Primary cultures of rat brain endothelial cells, glia, and pericytes, and construction of the in vitro blood–brain barrier (BBB) model were prepared according to the method in our previous studies [23,33]. For the establishment of the BBB model, pericytes were passaged at 1.5 × 10^4^ cells / cm^2^ density to the bottom side of 12-well tissue culture inserts (Transwell^®^, polycarbonate membrane, 0.4 µm pore size, Millicell, Merck, Germany). After attachment of pericytes, endothelial cells at the density of 7.5 × 10^4^ were seeded to the upper side of the membranes. Culture inserts were placed in 12-well plates containing glial cells with endothelial culture medium in both compartments. After three days of co-culture, when brain endothelial cells reached confluency and made tight barrier based on TEER measurements, we used the endothelial cells for permeability experiments. 

### 4.5. Measurement of the Integrity of the Paracellular Barrier

Transendothelial electrical resistance (TEER), reflecting the tightness of the interendothelial junctions, was measured by an EVOM Volt-ohmmeter (World Precision Instruments, Sarasota, FL, USA) combined with STX2 electrodes, and expressed relative to the surface area of the monolayers (Ω cm^2^). Resistance of cell-free inserts (100 Ω cm^2^) was subtracted from the measured values. The BBB model based on primary rat cells showed a TEER value of 292 ± 23 Ω cm^2^ (n = 10), indicating good barrier properties for the permeability assay. The hCMEC/D3 monolayer showed a TEER value of 53 ± 15 Ω cm^2^ (n = 40) before the experiments, indicating weaker barrier properties compared to primary rat brain endothelial cells.

### 4.6. Visualization of the Uptake of FITC-HPBCD in Brain Endothelial Cells

To visualize the cellular uptake of the FITC-HPBCD, primary rat brain endothelial cells were grown on glass-bottom Petri dishes (Greiner Bio-One, Monroe, NC, USA) coated with collagen IV and fibronectin and treated with 50 µM FITC-HPBCD for 2 or 24 h. To stain cell nuclei, Hoechst dye 33342 (1 μg/mL; 10 min) was used. After incubation, living cells were washed three times with Ringer–Hepes buffer (118 mM NaCl, 4.8 mM KCl, 2.5 mM CaCl_2_, 1.2 mM MgSO_4_, 5.5 mM D-glucose, 20 mM Hepes, pH 7.4) supplemented with 1% PDS, and examined with a confocal laser scanning microscope (Olympus Fluoview FV1000, Olympus Life Science Europa GmbH, Hamburg, Germany). 

The cellular uptake experiment on hCMEC/D3 cells was performed as follows: 1 × 10^5^ cells/well were seeded on round glass cover-slips placed into 24-well plates. Two days later, cells were washed once with Hank’s Balanced Salt Solution (HBSS) and incubated for 2 h or 24 h at 37 °C with 50 µM FITC-HPBCD solution. After incubation, cells were washed three times with HBSS and fixed with 3.7% paraformaldehyde solution for 15 min at room temperature. After fixation, cells were washed three times with HBSS and cell nuclei were stained with 4′,6-diamidino-2-phenylindole (DAPI; 283 nM) for 10 min at room temperature. The cells were washed once with HBSS and the round glass cover-slips were mounted to glass microscope slides. Fluorescence microscopy measurements and analyses were carried out by a Zeiss Axioscope A1 microscope (Jena, Germany). The following filters were used to examine the samples: DAPI: excitation G 365 nm, emission BP 445/50 nm; fluorescein: excitation BP 470/40 nm, emission BP 525/50 nm.

### 4.7. Immunostaining of Claudin-5 in Brain Endothelial Cells

To see the effect of FITC-HPBCD on integral membrane junctional protein claudin-5, primary rat brain endothelial cells were cultured on collagen IV and fibronectin-coated glass cover slips. After 50 µM HPBCD treatment for 2 or 24 h, cells were fixed with ethanol–acetic acid (95:5 *v*/*v*) for 5 min, blocked with 3% bovine serum albumin diluted in PBS, and incubated overnight with primary antibody anti-claudin-5 (rabbit polyclonal antibody, 1:200, AB_10753223). Incubation with secondary antibody Cy3-labeled anti-rabbit IgG, and Hoechst dye 33342, for nucleus staining, lasted for 1 h. Cells were washed three times with PBS between incubations. After mounting the samples (Fluoromount-G; Southern Biotech, Birmingham, AL, USA), staining was visualized by Olympus Fluoview FV1000 confocal laser scanning microscope (Olympus Life Science Europe GmbH, Hamburg, Germany).

hCMEC/D3 cells were seeded onto sterile collagen-coated glass microscope cover slides at a density of 5 × 10^4^ cells/slide and cultured until the formation of monolayers. Cells were washed with Hank’s Balanced Salt Solution (HBSS) three times and fixed with acetone/methanol 1:1 for 10 min at 4 °C. Cells were washed with HBSS three times and incubated in fetal bovine serum (FBS) for 30 min at room temperature to block the unspecific binding sites. Samples were washed again with HBSS three times, and primary staining was accomplished using mouse anti-human claudin-5 antibodies (1:200 dilution). After washing with HBSS three times, secondary labelling with Alexa Fluor 488 conjugated goat-anti-mouse IgG (1:400 dilution) was performed. Cell nuclei were stained with bis-benzimide and the samples were mounted on glass microscope slides for analysis. All antibodies were from Thermo Fisher Scientific (Waltham, MA, USA). For TJ protein staining, samples were observed by Zeiss Axio Scope.A1 fluorescence microscope (HBO 100 lamp) (Carl Zeiss Microimaging GmbH, Gottingen, Germany). Images were analyzed with ZEN 2012 v.1.1.0.0. software (Carl Zeiss Microscopy GmbH, Gottingen, Germany).

### 4.8. Permeability Measurements

The permeability of the FITC-HPBCD across the primary-cell-based BBB model was measured in the apical-to-basolateral (A–B, blood-to-brain) and basolateral-to-apical (B–A, brain-to-blood) directions at 37 °C in permeability buffer (phenol red free DMEM/F12 medium supplemented with 1% PDS, 1% ITS, and 1% HEPES) [22]. In the A–B permeability assay, cell culture medium was changed in the lower compartment of the inserts to 1500 µL permeability buffer. In the upper (donor) compartment, medium was replaced by permeability buffer containing 50 µM of FITC-HPBCD. In the case of the B–A permeability direction, cell culture medium was changed in the lower compartment of the inserts to 1500 µL permeability buffer containing 50 µM of cyclodextrin, and the upper (receiver) compartment medium was replaced by 500 µL permeability buffer. To check the monolayer integrity inserts with the BBB model were incubated with 10 µM sodium fluorescein (SF, 376 Da) and Evans blue-albumin (EBA, 67 kDa; 167.5 µg/mL and 1 mg/mL, respectively) passive hydrophilic permeability marker molecules. The culture plates were kept in a 37 °C incubator with 5% CO_2_ for 120 min on a rocking platform. Permeability measurement was also performed on cell-free inserts to test the cyclodextrin passage across the membrane alone. After 120 min incubation, samples from all compartments were collected and the concentration of FITC-HPBCD and the marker molecules were determined with spectrofluorometer (Fluorolog 3, Horiba Jobin Yvon, Edison, NJ, USA).

The apparent permeability coefficients (P_app_) of the test compounds were calculated as we described previously **[28]**. Briefly, cleared volume was calculated from the concentration of the tracer in the acceptor compartment ([C]_A_) after 120 min (t) and donor compartments at 0 h ([C]_D_), the volume of the acceptor compartment (V_A_; 1.5 or 0.5 mL), and the surface area available for permeability (A; 1.1 cm^2^) by the following equation:

(1) $$P_{\mathrm{app}}\;(\mathrm{cm}/s)\;=\frac{{\left[C\right]}_{A}\;\times \;V_{A}}{A\times {\left[C\right]}_{D}\times t}$$

For permeability measurements, hCMEC/D3 cells (2.5 × 10^5^ cells /insert) were seeded on culture inserts (Corning Costar 3401 Transwell^®^, polycarbonate membrane, 0.4 µm pore size, Millicell, Merck, Germany). ENDOGRO-MV culture medium was changed every 2–3 days on the cells. The model was used two weeks after seeding. The integrity of the monolayer was monitored by TEER measured with a Millicell–ERS volt-ohmmeter (Millipore, Billerica, MA, USA). In the case of hCMEC/D3 cell line, the permeability measurements were performed with the same protocol as in the case of primary rat brain endothelial cells with the following differences: cells were kept in ENDOGRO-MV culture medium, and SF, FITC-HPBCD, and FITC-dextran (4 kDa) solutions were used (1 mM stock solutions in HBSS were diluted in ENDOGRO-MV culture medium to a final concentration of 50 µM). Samples were collected from the donor compartment at 0 and 120 min and from the acceptor compartment at 30, 60, and 120 min (100 µL sample volumes were always replaced with ENDOGRO-MV). In permeability experiments, TEER values were also measured at the beginning and at the end of sampling to check monolayer integrity and follow the effects of cyclodextrin treatments. 

### 4.9. Uptake of FITC-HPBCD by hCMEC/D3 Cells, a Flow Cytometric Analysis

For flow cytometric measurement, hCMEC/D3 cell suspension was used (detached cells were washed two times with HBSS). The cell concentration was set to 1 × 10^6^ cells/mL using HBSS. In each experiment, the cellular fluorescence of untreated control cells, 50 µM FITC-HPBCD treated at 37 °C for 30 min, and 50 µM FITC-HPBCD treated on ice for 30 min was measured in duplicates. After treatment, samples were washed three times with ice-cold HBSS, kept on ice, and propidium iodide (1 µg/mL) was added to exclude dead cells. Measurement was made with Guava easyCyte HT flow cytometer (Merck, Darmstadt, Germany). GreenB and RedB channels were used to analyze the cellular fluorescence. Three or four independent experiments were carried out.

### 4.10. Investigation of the Lysosomes

#### 4.10.1. Fluorescence Microscopy

Fluorescence microscopy investigation of lysosomes was carried out as described earlier on Caco-2 cells [15]. hCMEC/D3 cells (5 × 10^4^ cells/well) were cultured on round glass coverslips placed into 24-well plates. Three days later, cells were washed once with HBSS and incubated for 30 min or 2 h at 37 °C with 50 µM FITC labeled or unlabeled HPBCD solutions. In the last 30 min of experiment, LysoTracker^®^ fluorescent reagent at 50 nM was added to the samples. Then, cells were washed three times with HBSS and fixed with 3.7% formaldehyde solution for 15 min at room temperature. After fixation, cells were washed three times with HBSS, and cell nuclei were stained with DAPI (283 nM) for 10 min at room temperature. Cells were washed once with HBSS and the round glass cover-slips were glued to the slides. Fluorescent microscopy measurements and analyses were carried out by a Zeiss Axioscope A1 (Carl Zeiss AG, Jena, Germany) fluorescent microscope. The following filters were used to examine the samples: DAPI: excitation G 365 nm, emission BP 445/50 nm; fluorescein: excitation BP 470/40 nm, emission BP525/50 nm; rhodamine: excitation BP 546/12 nm, emission BP 575–640 nm.

#### 4.10.2. Flow Cytometry

In this experiments, FITC-HPBCD and HPBCD were used similarly to the earlier investigations [15]. hCMEC/D3 cell suspension was prepared by trypsinization of cells with 0.05% trypsin–EDTA solution, washed twice with HBSS, and the cell concentration was set to 1 × 10^6^ cells/mL. Cells were pre-incubated with 50 µM cyclodextrin solutions for 30 min or 2 h at 37 °C. For the last 30 min of incubation, 50 nM LysoTracker^®^ reagent was added to the cells. After incubation time, cells were washed twice with ice-cold HBSS and fixed with 1% PFA. Cellular fluorescence was analyzed by Guava Easy Cyte 6HT-2L flow cytometer (Merck Ltd., Darmstadt, Germany). FITC-labeled cyclodextrins were analyzed by using 488 nm excitation and 525/30 nm emission wavelengths (green channel), while LysoTracker was measured at 695/50 nm.

### 4.11. Statistics

Data are presented as means ± SEM. Values were compared using one-way ANOVA following Dunnett post-tests (GraphPadPrism 5.0 and 7.0; GraphPad Software, (GraphPad Software Inc., San Diego, CA, USA). Changes were considered statistically significant at *p* < 0.05. The number of parallel samples was 4–8. 

## Data Availability

Not applicable.
